# Experimental and explainable machine learning approach on thermal conductivity and viscosity of water based graphene oxide based mono and hybrid nanofluids

**DOI:** 10.1038/s41598-024-81955-1

**Published:** 2024-12-28

**Authors:** Praveen Kumar Kanti, Prabhu Paramasivam, V. Vicki Wanatasanappan, Seshathiri Dhanasekaran, Prabhakar Sharma

**Affiliations:** 1 Institute of Power Engineering, Universiti Tenaga Nasional, Jalan IKRAM-UNITEN, Selangor, 43000 Malaysia; 2 University Centre for Research & Development (UCRD), Chandigarh University, Mohali, 140413 Punjab India; 3 Centre for Research Impact & Outcome, Chitkara University Institute of Engineering and Technology, Chitkara University, Rajpura, 140401 Punjab India; 4 Department of Computer Science, UiT The Arctic University of Norway, Tromso, 9037 Norway; 5 Department of Mechanical Engineering, Delhi Skill and Entrepreneurship University, Delhi, 110089 India; 6 Department of Mechanical Engineering, Mattu University, Mettu, 318 Ethiopia

**Keywords:** Graphene oxide, Hybrid nanofluid, Regression, Explainable ML, Titanium dioxide, Mechanical engineering, Fluid dynamics

## Abstract

This study explores the thermal conductivity and viscosity of water-based nanofluids containing silicon dioxide, graphene oxide, titanium dioxide, and their hybrids across various concentrations (0 to 1 vol%) and temperatures (30 to 60 °C). The nanofluids, characterized using multiple methods, exhibited increased viscosity and thermal conductivity compared to water, with hybrid nanofluids showing superior performance. Graphene oxide nanofluids displayed the highest thermal conductivity and viscosity ratios, with increases of 52% and 177% at 60 °C and 30 °C, respectively, for a concentration of 1 vol% compared to base fluid. Similarly, *graphene oxide-TiO*_*2*_ hybrid nanofluids achieved thermal conductivity and viscosity ratios exceeding 43% and 144% compared to the base fluid at similar conditions. This data highlights the significance of nanofluid concentration in influencing thermal conductivity, while temperature was found to have a more pronounced effect on viscosity. To tackle the challenge of modeling the thermophysical properties of these hybrid nanofluids, advanced machine learning models were applied. The Random Forest (RF) model outperformed others (Gradient Boosting and Decision Tree) in both the cases of thermal conductivity and viscosity with greater adaptability to handle fresh data during model testing. Further analysis using shapely additive explanations based on cooperative game theory revealed that relative to temperature, nanofluid concentration contributes more to the predictions of the thermal conductivity ratio model. However, the effect of nanofluid concentration was more dominant in the case of viscosity ratio model.

## Introduction

Nanofluids, composed of nanoparticles dispersed within a base fluid, have gained prominence in thermal system research due to their exceptional capability to enhance heat transfer properties, particularly through greater thermal conductivity^1^. These advanced colloidal mixtures have captured the interest of researchers for their potential to significantly boost thermal performance, making them suitable for a wider range of industrial thermal applications^2^. The nanoparticles suspended in the fluid matrix play a significant role in improving heat transfer efficiency. Moreover, the flexibility to tailor nanofluid properties by adjusting concentration and temperature allows for precise customization to meet the demands of specific applications^3^. This flexibility highlights the potential of nanofluids to improve system efficiency and contribute to more sustainable thermal management technologies, opening new avenues for innovation in heat transfer systems and energy-saving initiatives^4^.

Hybrid nanofluids represent a cutting-edge development in thermal management systems, consisting of a combination of different nanoparticles suspended in a base fluid. This innovative strategy merges the distinct properties of various nanoparticles, creating a synergistic effect that leads to superior thermal performance compared to traditional single-component nanofluids^5,6^. The primary advantage of hybrid nanofluids lies in their ability to capitalize on the complementary attributes of diverse nanoparticles. By strategically selecting materials with varying shapes, sizes, and thermal characteristics, researchers can design fluids with enhanced heat transfer properties and increased stability. This combination often results in non-linear improvements in specific heat capacity, thermal conductivity, and viscosity, outperforming single-component nanofluids^7,8^.

The adaptability of hybrid nanofluids enables them to be tailored for specific application needs. For example, combining metallic and non-metallic nanoparticles can help achieve a balance between high thermal conductivity and long-term stability^9,10^. Likewise, integrating carbon-based nanostructures with ceramic particles could produce a nanofluid with superior heat transfer capabilities and improved mechanical strength. Optimizing the performance of hybrid nanofluids in thermal management applications requires a thorough understanding of their transport phenomena^11^. This involves a detailed analysis of their rheological properties, thermal conductivity, viscosity, convective heat transfer, stability and dispersion, along with the effects of Brownian motion and thermophoresis.

By examining these properties and their interactions, researchers can create predictive models for the behavior of hybrid nanofluids. This understanding is crucial for designing more efficient cooling systems for electronics, enhancing industrial heat exchangers, improving solar thermal collectors, and advancing energy storage solutions^12–14^. Additionally, studying hybrid nanofluids expands our fundamental knowledge of nanoscale fluid dynamics and interfacial phenomena. This deeper comprehension not only helps refine current technologies but also encourages innovation in areas like nanofluidics, colloidal science, and advanced materials engineering^15,16^. As research continues, hybrid nanofluids are expected to play a key role in overcoming thermal management challenges in next-generation technologies, potentially resulting in more energy-efficient and compact cooling solutions for various industries. Recently, many researchers have been exploring ternary nanofluids for thermal management applications^17–19^.

When combined, graphene oxide *(GO)*, metal oxides such as silicon dioxide *(SiO*_*2*_*)*, and titanium dioxide *(TiO*_*2*_*)* create an effective solution for advanced heat transfer applications. GO’s outstanding TC and greater surface area promote efficient heat conduction and enhance surface contact. *TiO*_*2*_, with its high refractive index and excellent thermal stability, effectively scatters and absorbs light, especially in ultraviolet-based systems, improving heat transfer efficiency. *SiO*_*2*_, renowned for its superior insulation and chemical resistance, helps reduce heat loss and ensures the long-term stability of the system. This synergistic combination provides considerable advantages in different domains of applications, including heat exchangers, thermal management systems, and insulation materials. By harnessing the distinct properties of these materials, researchers and engineers can develop cutting-edge solutions to meet the increasing demand for efficient and sustainable thermal management. Table 1 highlights recent investigations on the thermophysical characteristics of GO-based nanofluids.

**Table 1:** Thermophysical properties of graphene oxide based nanofluids.

Author	Nanofluid	Concentration	Temperature (^o^C)	Remarks
Mei et al.^20^	*GO*/water	0.002 to 0.01 mass %	25–50	Highest thermal conductivity obtained for a concentration of 0.01% at 50 °C
Selvem et al.^21^	*GO*/Ethylene glycol-water	0-0.45 vol%	30	Highest thermal conductivity augmentation of 18% at 0.45 vol%.
Yadav et al.^22^	*GO*/Ethylene glycol	0-0.25 mass %	10–50	Thermal conductivity enhancement of 36.72% noticed.
Esfahani and Languri^23^	*GO*/water	0.01–0.1 mass %	25–40	Viscosity increment of 60% for 0.1 wt% at 25 ^o^C.
Ranjbarzadeh et al.^24^	*GO-SiO*_*2*_	0–1 vol%	20–60	Viscosity of the hybrid nanofluid of 345% compared to the base fluid.
Kanti et al.^25^	*GO-Al*_*2*_*O*_*3*_ (50:50)/water	0–1 vol%	30–60	maximum TC enhancement of GO is 43.9% higher than Al_2_O_3_ nanofluid at 1 vol% at a temperature of 60 °C
Kanti et al.^26^	*GO-CuO* (50:50 and 80:20)/water	0–1 vol%	30–60	Viscosity and thermal conductivity of GO/CuO (50:50) HNF are higher than that of GO/CuO (20:80)
Kanti et al.^27^	*GO-MXene* (50:50)/water	0–1 vol%	25–60	Addition of MXene to GO NF reduces the thermal conductivity of GO nanofluid.
Selvarajoo et al.^28^	*Al*_*2*_*O*_*3*_*-GO* (80:20)/water	0–1 vol%	30–50	Maximum thermal conductivity enhancement of hybrid nanofluid was about 4.3 and 4.34% greater than Al_2_O_3_ and GO mono nanofluid
Huminic et al.^29^	*GO-Si*	0.25 wt%	20–50	Viscosity increment due to larger specific surface area of GO sheets.
Colak et al.^30^	*Al*_*2*_*O*_*3*,_*Cu*/water	0-0.2 vol%	25–65	Al_2_O_3_/water has the highest specific heat capacity compared to Cu nanofluid
Colak et al.^31^	*Cu-Al*_*2*_*O*_*3*_/water	0-0.2 vol%	20–65	Specific heat capacity of nanofluid improves with increase in temperature.

This research delves into the thermophysical properties of water-based nanofluids containing *GO*, *SiO*_*2*_, *TiO*_*2*_, and their hybrid combinations (50:50) across various concentrations and temperatures. Unlike previous studies focused on individual nanomaterial’s, this investigation uniquely examines hybrid nanofluids, illuminating their potential to enhance both thermal conductivity and viscosity. The study employs cutting-edge modeling techniques, particularly machine learning algorithms, to accurately predict these intricate properties. Key questions addressed include the comparative performance of hybrid nanofluids versus single-component alternatives, the precision of advanced machine learning models like extreme gradient boosting and deep neural networks in predicting thermophysical characteristics, and the crucial factors influencing nanofluid behavior, as revealed through analytical methods such as SHAP. This work bridges a significant research gap by elucidating the superior performance of hybrid nanofluids and leveraging sophisticated machine learning models to gain profound insights into the determinants of their thermophysical properties.

As a first step, the study involved synthesizing and characterizing *GO*,* SiO*_*2*_, and *TiO*_*2*_ nanoparticles, followed by the formulation of nanofluids with concentrations ranging from 0 to 1 vol%. The stability of these nanofluids was assessed, and their thermal conductivity and viscosity were determined at temperatures ranging from 30 to 60 °C. Furthermore, machine learning approaches were used to characterize the behavior of nanofluids based on thermal conductivity and viscosity data from all tested nanofluids.

## Methodology

### Synthesis of nanoparticles, characterization, and Nanofluid Preparation

The chemical reagents used in this study were obtained from Merck and employed without additional purification. Deionized water had been used consistently throughout the test process. Details on the synthesis of the required nanoparticles can be found in the associated paper^32^. To evaluate the stability of the nanofluids, a Malvern Instruments, UK make Nano-ZS apparatus was utilized. The structural and morphological characteristics of the nanoparticles were analyzed using X-ray diffraction and field emission scanning electron microscopy. Water-based mono and hybrid nanofluids in a 50:50 ratio were produced using a two-step method. For information on the preparation of the nanofluids, please refer to our published article^32^.

### Thermal conductivity and viscosity

The thermal conductivity of the nanofluids was measured using a KD2 Pro Thermal Properties Analyzer from Decagon Devices Inc. This device, featuring a single KS-1 sensor, employs the transient hot-wire method. The sensor, which is 60 mm long and 1.3 mm in diameter, is immersed in the test fluid and functions as both a heat source and a temperature sensor. The transient hot-wire method minimizes issues with free convection by producing a small amount of heat. The instrument has a maximum error margin of ± 5%. Before starting the experiments, the KD2 Pro was calibrated with a glycerol standard provided by the manufacturer.

The viscosity of the nanofluids was assessed employing a Brookfield DV-II + Pro viscometer with maximum uncertainty of ± 2%. This instrument measures fluid resistance by evaluating the torque exerted by a spindle submerged in the fluid. To ensure accurate readings, a controlled water bath was used alongside the viscometer to maintain temperature stability and assess the impact of temperature on viscosity. Viscosity measurements were taken at temperatures ranging from 30 to 60 °C in 5 °C increments. Each measurement was repeated five times for all concentrations and temperatures to ensure consistency and reliability, with a 15-minute interval between each measurement. Please refer our published article^32^ for the validation of both the instruments.

### Prognostic analysis with machine learning

#### Random forest

Random Forest (RF) is an ensemble learning method that operates by constructing multiple decision trees during training and outputting either the mode of classification or the mean prediction for regression tasks. Within the framework of regression, the model averages the outputs of individual decision trees^33–35^. Every decision tree reduces overfitting and variance by being constructed using a random subset of the data and a random subset of the features at every node. The technique randomly chooses samples with replacement—bootstrapping—during training to ensure that each tree is trained on another subset. Random feature splitting guarantees that every tree is decorrelated, therefore strengthening the whole ensemble model^36,37^. Computed as the average of all tree forecasts, the final prediction simultaneously reduces bias and variation. Especially in datasets where decision trees by themselves may show great variance, this approach usually proves strong against overfitting. Because of the averaging of several trees, the training error is typically minimal; nonetheless, overfitting on training data subsets can cause a modest increase in the test error, but less clearly than in single decision trees^38,39^.

#### Gradient boosting regression

Gradient Boosting (GB) Regression builds models sequentially, where each new model corrects the errors of the previous one. Usually mean squared error for regression problems, it is a stage-wise additive model adding new trees to minimize the loss function. The procedure begins with training an original data-based decision tree, then computes residuals or errors from this tree^40,41^. One then trains a fresh tree to forecast these residuals. This tree is included into the model and its forecasts are merged with the past trees to change the general prediction of the model. This procedure is repeated for a designated number of times, with every next tree fixing the errors of the last one^42,43^. Gradient descent helps to minimize a differentiable loss function, hence updating the model parameters. Though it can be prone to overfitting, especially when too many iterations are carried out, gradient boosting is renowned for its great predictive ability. Its iterative character of concentrating on the most challenging samples at each stage makes it usually more accurate than decision trees and random forests^44,45^.

#### Decision tree

Decision trees (DT) operate by recursively partitioning the data into subsets based on the values of input features, creating a tree-like model of decisions. At every node, the method chooses the feature and threshold that reduces a given loss function, like mean squared error in regression problems. Until the data is sufficiently partitioned or a stopping criterion—like the maximum depth—this method iteratively generates branches in the tree. Without feature scaling or data normalizing, decision trees can record intricate relationships among characteristics^46^. Average of the target values in every leaf node generates the last forecast. Since the framework of decision trees reflects human decision-making procedures, they are straightforward to understand^47,48^. They do, however, overfit easily, particularly in cases whereby the trees grow deep and capture noise in the training data. Pruning helps to minimize the overfitting issue by simplifying the model by eliminating branches with minimal predictive capability. Although decision trees are strong, their single-tree architecture increases their sensitivity to high variance relative to ensemble techniques like random forests or gradient boosting^49,50^.

#### SHAP-based explainable machine learning

SHapley Additive exPlanations (SHAP) values are a game-theory-based approach to interpreting machine learning models by quantifying the contribution of each feature to the model’s output. SHAP values are especially helpful in clarifying how thermal conductivity and viscosity of water-based graphene oxide nanofluids are influenced by variables like nanoparticle concentration, temperature, and other experimental circumstances^51,52^. By computing the contribution of every characteristic to every individual prediction, SHAP provides insights on the internal dynamics of complicated machine learning models, hence explaining their output. Using random forest (best performing in this study), the researchers would train machine learning models in this work to predict the TC and viscosity employing experimental data. SHAP values for every feature would be computed following training so that the researchers may observe how factors such as temperature or concentration of nanoparticles affect the expected thermal characteristics^53,54^. Summary plots help one see SHAP values; each point in these graphs represents a prediction and the size of the SHAP values indicates the contribution of a feature. This makes it possible to clearly grasp the fundamental physics and data patterns, including whether and by what degree raising nanoparticle concentration affects heat conductivity or viscosity. SHAP values’ explainability gives the machine learning predictions a fresh layer of transparency, therefore enabling domain experts to better understand the outcomes^55,56^.

## Result and discussion

### Characterization

**Fig. 1:**
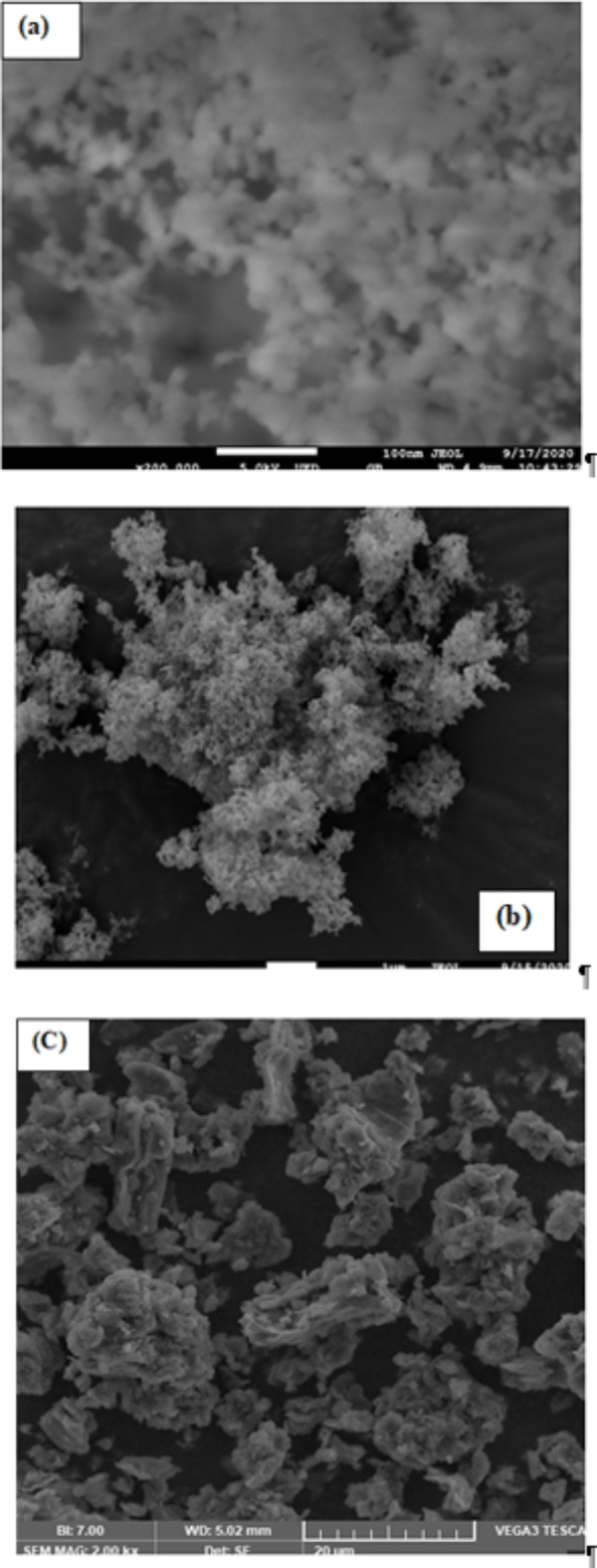
FESEM images for (**a**) SiO_2_ (**b**) TiO_2_ and (**c**) GO.

**Fig. 2:**
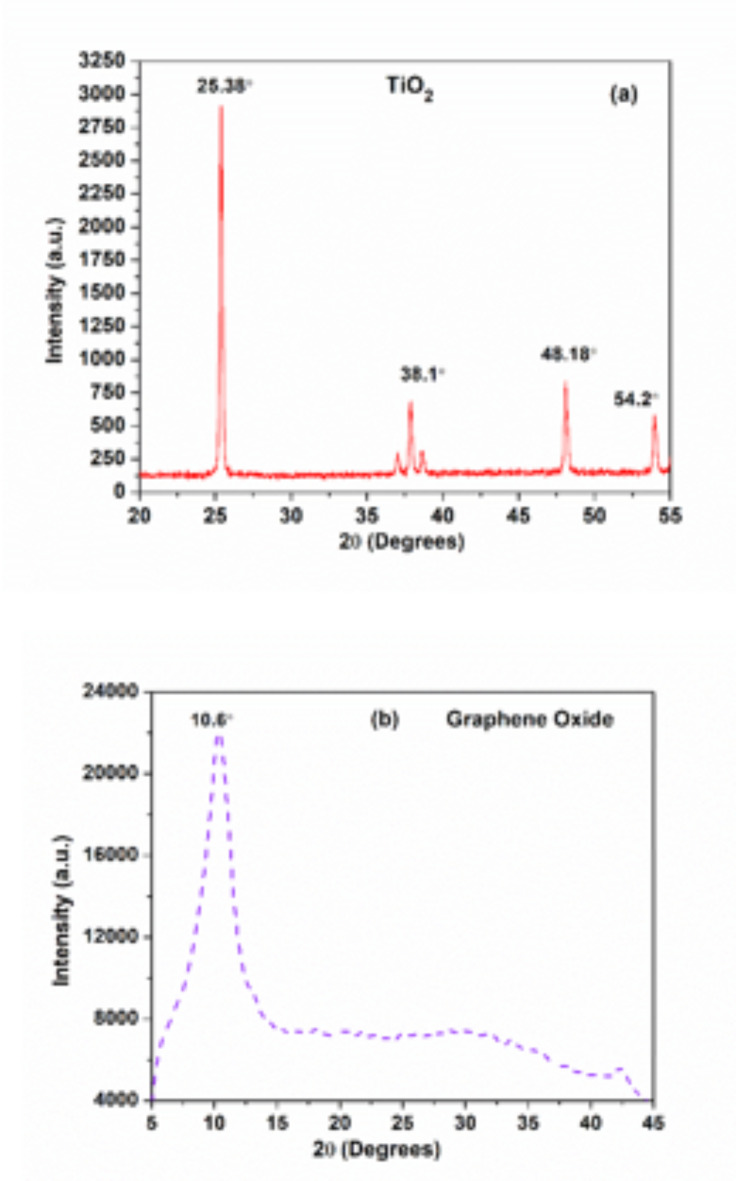
XRD for (**a**) *TiO*_*2*_ and (**b**) *GO* nanoparticle.

**Fig. 3:**
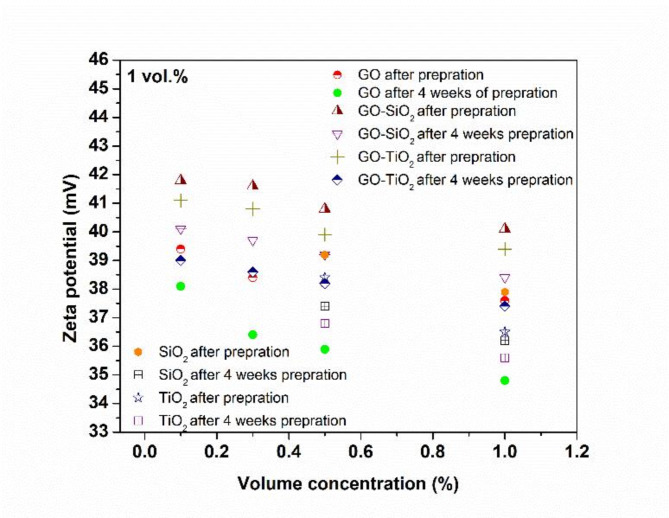
Zeta potential values for considered nanofluids.

Figure 1a to c show field emission SEM images of the nanoparticles (Fig. 2). Analysis of Fig. 3a and b indicates that both *SiO*_*2*_ and *TiO*_*2*_ nanoparticles have a spherical shape and tend to aggregate due to Van der Waals forces. This spherical form is beneficial because it improves the contact surface area between the nanoparticles and the base fluid, hence improving heat transfer efficiency. Additionally, the uniformity of spherical particles helps reduce fluid resistance, promoting smoother flow dynamics in nanofluids. In contrast, Fig. 1c illustrates that *GO* nanoparticles have a layered or sheet-like structure. Unlike spherical particles, the sheet-like structure of *GO* may increase the viscosity of the nanofluid, potentially affecting its flow characteristics. However, the high surface area of these *GO* nanosheets can enhance the stability and thermal conductivity of the nanofluid, offsetting the potential viscosity increase. Furthermore, the spherical shape of metal oxide nanoparticles helps lower the viscosity of the nanofluids in comparison with irregular structures like *GO*. This makes *SiO*_*2*_ and *TiO*_*2*_ more suitable for applications where it is crucial to maintain low viscosity while improving heat transfer. Combining these materials can provide a balance between enhanced thermal conductivity and manageable viscosity levels in hybrid nanofluid systems.

The nanoparticles were characterized using a Bruker Advance diffractometer with *Cu-Kα* radiation over a *2θ* range of 10° to 60°, at a 0.02° step size. The crystallite size was calculated using the Scherrer formula. XRD analysis of *TiO*_*2*_ (Fig. 3a) shows pure anatase phases, confirming no impurities and a mean nanoparticle size of 40 nm. In XRD analysis presented in Fig. 3b, *GO* typically shows a peak around *2θ = 10–15°* due to the increased interlayer spacing caused by oxygen functional groups. This peak confirms the successful oxidation of graphite into *GO*.

### Nanofluid stability

The zeta potential is an important parameter in determining the dispersed nanoparticles stability in nanofluids. A zeta potential value exceeding ± 30 mV indicates stable colloidal dispersions.

In this study, the mass ratio of *Polyvinyl pyrrolidone (PVP)* to *GO*, and to hybrid nanofluids, was kept constant and matched the weight of *GO* to ensure consistent experimental conditions for accurate comparisons. Stability analysis using the Zetasizer (Malvern Instruments, UK) revealed that zeta potential values were consistent both immediately after preparation and after 25 days, demonstrating the high stability of the nanofluids shown in Fig. 3. These results highlight the effective dispersion of nanoparticles, which is essential for enhancing heat transfer and ensuring long-term stability and performance.

### Viscosity

**Fig. 4:**
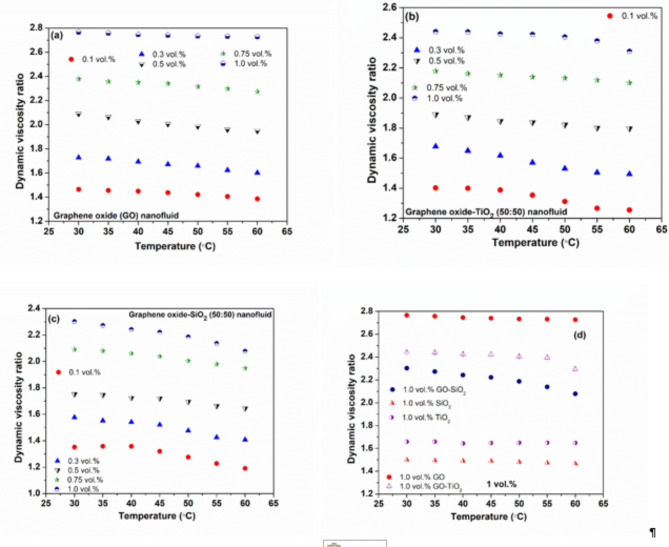
Depiction of dynamic viscosity ration in the case of (**a**) GO (**b**) GO-TiO_2_ (**c**) GO-SiO_2_ hybrid NF, (**d**). Comparative depiction of considered nanofluids dynamic viscosity ratio.

Figure 4a–d illustrate how the viscosity of nanofluids varies with concentration and temperature. Viscosity rises with increased concentration but decreases as temperature increases. The highest and lowest viscosity ratio (µ _nf_/µ _bf_) observed for GO nanofluid is 2.77 and 1.38 at 30 and 60 °C for 1 and 0.1 vol%, correspondingly. The relationship between temperature and viscosity in nanofluids is governed by a complex interplay of molecular dynamics and nanoparticle behavior. As thermal energy increases, it catalyzes more vigorous molecular motion, weakening the cohesive forces between fluid molecules. This diminution of intermolecular attraction facilitates easier relative movement, manifesting as reduced viscosity and enhanced flow characteristics. Concurrently, elevated temperatures amplify the Brownian motion of suspended nanoparticles, causing them to collide more frequently with fluid molecules and disrupt local fluid structures. This disruption further attenuates fluid viscosity by impeding the formation of transient molecular networks that contribute to flow resistance.

However, the influence of nanoparticles on viscosity is concentration-dependent and non-linear. At higher concentrations, nanoparticles exhibit increased propensity for inter-particle interactions, potentially leading to the formation of temporary or permanent agglomerates. These larger structures can significantly impede fluid flow, counteracting the viscosity-reducing effects of temperature. Moreover, the surface properties of nanoparticles, such as charge distribution and functional groups, play a crucial role in determining their aggregation behavior and subsequent impact on fluid dynamics.

The presence of nanoparticles also introduces additional mechanisms that modulate viscosity. For instance, the formation of a nanolayer of fluid molecules around each particle can alter local fluid properties, creating regions of modified viscosity that influence bulk fluid behavior. Furthermore, the shape anisotropy of certain nanoparticles, such as *GO* flakes, can induce orientation-dependent effects on fluid flow, adding another layer of complexity to the viscosity profile of nanofluids at varying temperatures and shear rates.

Figure 4d contrasts at various concentrations and temperatures the viscosity of hybrid and mono nanofluids. Whereas SiO_2_ nanofluids display the lowest viscosity, GO nanofluids have the greatest. The plate-like structure and large surface area of *GO* hinder fluid movement, increasing its viscosity. On the other hand, the smaller surface area and the spherical shape of *SiO*_*2*_ facilitate easier fluid flow, resulting in lower viscosity. *TiO*_*2*_ nanofluids have higher viscosity than *SiO*_*2*_, likely due to their smaller particle size and higher density. *GO-TiO*_*2*_ nanofluids display higher viscosity compared to *GO-SiO*_*2*_, as *TiO*_*2*_’s smaller particles and SiO_2_’s shape promote smoother flow. Hybrid nanofluids, such as *GO-SiO*_*2*_ or *GO-TiO*_*2*_, generally exhibit higher viscosity than mono nanofluids due to increased particle interactions, larger particle clusters, and potential synergistic effects between different nanoparticles, leading to greater flow resistance. While adding GO raises viscosity, mixing it with *SiO*_*2*_ or *TiO*_*2*_, which have lower surface areas, reduces the viscosity of *GO* in hybrid formulations.

### Thermal conductivity

**Fig. 5:**
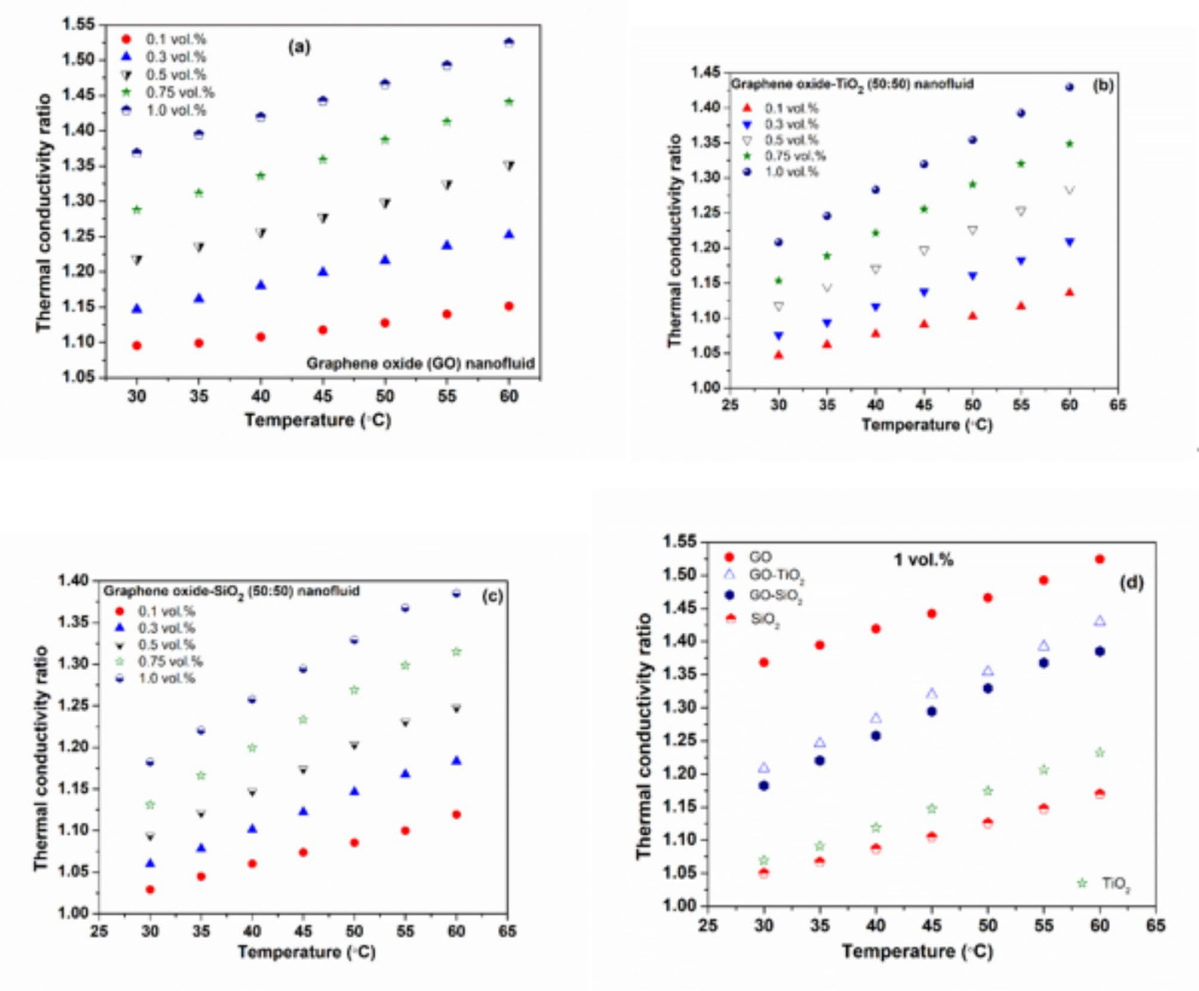
TC in the case of (**a**) GO (**b**) GO-TiO_2_ (**c**) GO-SiO_2_ hybrid NF, (**d**) Thermal conductivity of the nanofluids for 1 vol%.

Figure 5 (a–c) illustrate the thermal conductivity of both hybrid and mono nanofluids, which increases with temperature and concentration. The maximum and minimum thermal conductivity amplification of 1.52 and 1.09 noticed for *GO* nanofluid at 60 and 30 °C for 1 and 0.1 vol%, respectively, compared to the water.

The thermal conductivity of nanofluids exhibits a complex relationship with temperature and nanoparticle concentration, driven by several interacting mechanisms. As temperature rises, the intensified Brownian motion of nanoparticles leads to improved dispersion and reduced agglomeration, creating a more uniform distribution of heat-conducting elements throughout the fluid. This enhanced particle mobility facilitates more frequent and energetic collisions between nanoparticles and fluid molecules, establishing numerous transient nanoscale heat transfer bridges that significantly boost overall thermal conductivity. The augmented thermal energy at elevated temperatures also promotes the formation of more robust and extensive percolation networks among nanoparticles, particularly in fluids with higher concentrations. These networks serve as preferential pathways for rapid heat propagation, markedly enhancing the fluid’s thermal transport capabilities. Furthermore, the temperature-induced reduction in fluid viscosity allows for more efficient heat transfer at the nanoparticle-fluid interface, as the decreased resistance to molecular motion enables swifter energy exchange.

Increasing nanoparticle concentration introduces additional heat conduction pathways, amplifying the fluid’s thermal conductivity. However, this relationship is non-linear and exhibits a critical threshold. Beyond a certain concentration, particle crowding can lead to the formation of larger aggregates, which paradoxically may impede heat flow by reducing the effective surface area for heat exchange and disrupting the continuity of thermal pathways. The synergistic interplay between temperature and concentration effects on thermal conductivity is particularly noteworthy. Higher temperatures can mitigate the negative impacts of increased concentration by enhancing particle dispersion and preventing excessive agglomeration, thus maintaining optimal heat transfer conditions even at elevated particle loadings.

It’s crucial to consider the role of nanoparticle material properties in this context. Materials with high intrinsic thermal conductivity, such as graphene or carbon nanotubes, can yield disproportionate increases in fluid thermal conductivity even at relatively low concentrations. The aspect ratio and surface chemistry of nanoparticles also significantly influence their dispersion behavior and interfacial thermal resistance, further modulating the temperature and concentration-dependent thermal conductivity enhancements in nanofluids.

Figure 5d compares the TC of hybrid and mono NFs. At 1.0 vol% and 60 °C, the highest thermal conductivity ratios for *SiO*_*2*_, TiO_2_, *GO-SiO*_*2*_, *GO-TiO*_*2*_, and *GO* nanofluids are 1.17, 1.23, 1.39, 1.43, and 1.52 respectively.

*GO* demonstrates the most significant TC enhancement owing to its inherent higher TC and layered structure, while *SiO*_*2*_, with lower thermal conductivity and surface area, shows the least. Hybrid nanofluids generally outperform mono nanofluids (except for GO) due to synergistic effects that combine the properties of different nanoparticles to create more efficient heat transfer pathways. Various nanoparticles improve thermal conductivity through enhanced phonon transport, better dispersion, and reduced agglomeration. Additives also help improve dispersion, which enhances the thermal conductivity of hybrids. *GO-TiO*_*2*_ exhibits higher thermal conductivity than *GO-SiO*_*2*_, due to *TiO*_*2*_’s smaller size and higher thermal conductivity. The improvement in thermal conductivity for nanofluids depends on particle size, shape, dispersion, and inter-particle interactions, which are essential for effective thermal management.

### Data pre-analysis

**Fig. 6:**
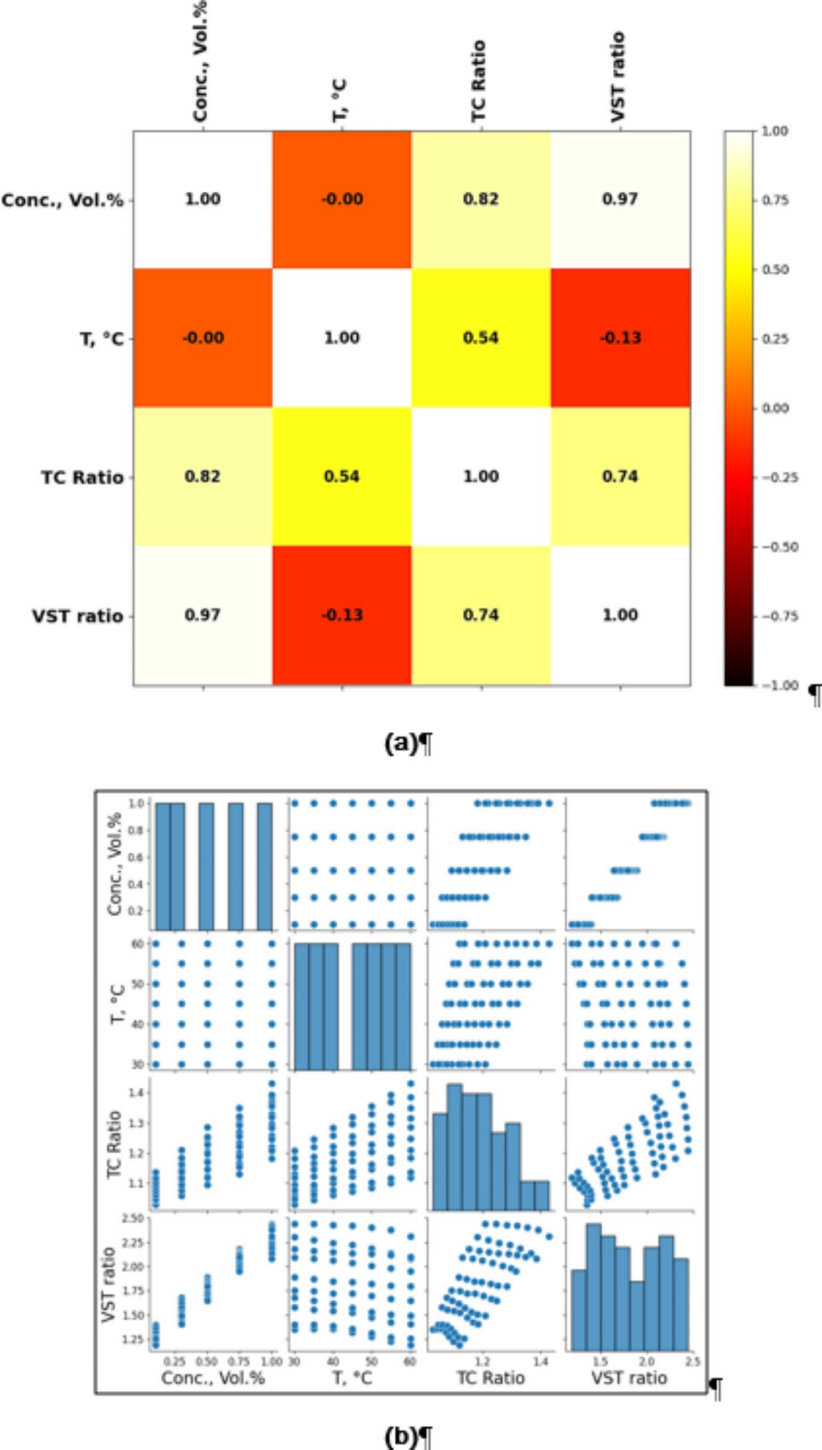
Correlations (**a**) Heatmap (**b**) pair plots developed using open access python libraries (pandas, matplotlib.pyplot, numpy).

The data gathered in lab-based testin phase was evaluated for correlation among the data columns. The correlation matrix offers important new perspectives on the interactions among the variables. Beginning with nanoparticle concentration, it shows a quite significant positive association with the viscosity ratio (0.97), meaning that the viscosity ratio also grows practically proportionately as the nanoparticle concentration rises (Fig. 6a**)**. This makes sense as the inclusion of nanoparticles usually raises the viscosity of the nanofluid. Likewise, the concentration displays a strong positive association with the thermal conductivity ratio (0.82), implying that increasing nanoparticle concentrations enhance the thermal conductivity of the fluid. Conversely, temperature (T) has no effect on concentration (0) since in this scenario the concentration of nanoparticles is independent of temperature. With the TC ratio (0.54), it does, however, exhibit a modest positive correlation, meaning that higher temperatures usually improve the TC of the nanofluids. By contrast, the temperature exhibits a mild negative association with the viscosity ratio (-0.13), meaning that rising temperature somewhat lowers viscosity, a normal behavior for fluids where viscosity drops with heating.

At last, the viscosity ratio and TC ratio show a quite strong positive connection (0.74), implying that in the nanofluid these two characteristics are connected. A rise in one generally follows a rise in the other as the nanoparticles raise both viscosity and heat conductivity. Understanding how changing one feature could affect the total thermal performance of the nanofluid depends on this interdependence. The scatter plot (pair plot) depicted in Fig. 6b visually shows the associations between several dataset variables. Whereas the off-diagonal parts display scatter plots, indicating the correlations between pairs of variables, each diagonal element provides a histogram for a single variable. This plot supports the results of the correlation matrix shown in Fig. 6a. While temperature has a secondary influence on thermal conductivity more than viscosity, the concentration of nanoparticles has a very significant effect on both thermal conductivity and viscosity ratios. The image demonstrates that concentration drives the behavior of the system mostly; temperature has a relatively moderate influence.

### Thermal conductivity ratio models

**Fig. 7:**
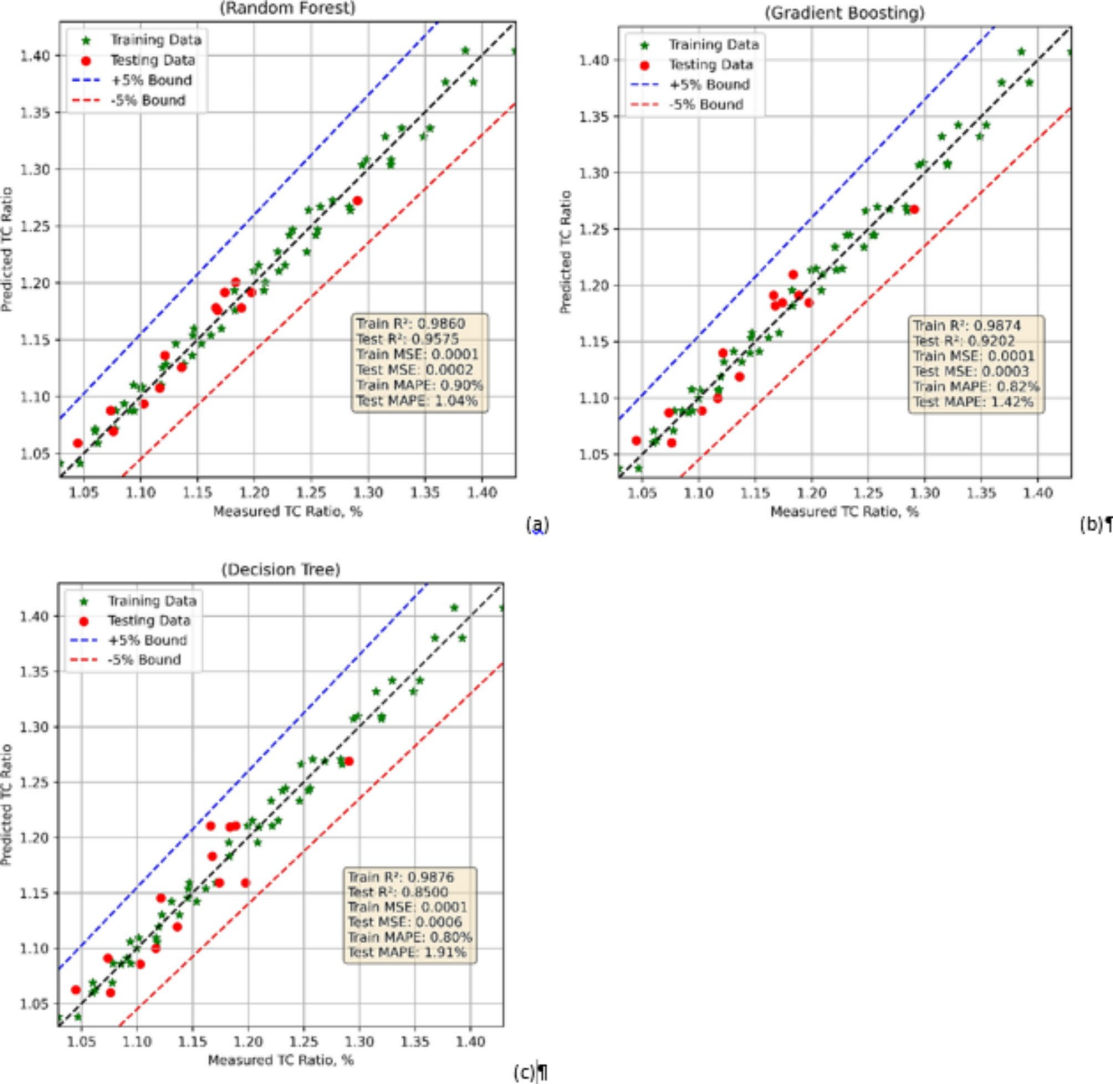
Model comparison in case of TC ratio model using (**a**) RF (**b**) GB (**c**) DT.

The TC ratio prediction models were developed by employing three modern ML approaches namely Random Forest (RF), Gradient Boosting (GB), and Decision Tree (DT). The models were then used for statistic-based evaluation and comparison. Table 2 shows the values of mean squared error (MSE), coefficient of determinants (R^2^), and mean absolute percentage error (MAPE) in each case. With a low Train MSE of 0.0001 and Test MSE of 0.0002 the RF model (Fig. 7a) exhibits good predictive ability. Train R² of 0.9860 and Test R² of 0.9575 show that the model generalizes effectively to unknown data, therefore attesting to great accuracy. Reflecting little prediction errors, the MAPE is likewise rather low, with 0.90% for training and 1.04% for testing. With a Train MSE of 0.0001 and Train R² of 0.9874 indicating outstanding fit the GB model (Fig. 7b) performs similarly on the training set. On the other hand, the Test MSE is somewhat higher at 0.0003 and the Test R² falls to 0.9202, therefore indicating less generalization than the Random Forest. With a MAPE for the test set of 1.42%, greater than that of Random Forest, this model clearly generates more prediction errors in unseen data. Reflecting a quite strong match to the training data, the DT model has a similar Train MSE of 0.0001 and a high Train R² of 0.9876. With a higher Test MSE of 0.0006 and Test R² of 0.8500, its performance on the test set does, however, clearly deteriorates. This suggests that the model suffers with generalizing. Furthermore, supporting that this model generates more errors while testing than Random Forest and Gradient Boosting is the test MAPE of 1.91%. While Decision Tree (Fig. 7c) displays symptoms of overfitting and less dependable predictions on the test data, Random Forest offers the best balance between training and test performance followed by Gradient Boosting.

**Table 2:** Model evaluations.

Model	Train MSE	Test MSE	Train *R*^2^	Test *R*^2^	Train MAPE, %	Test MAPE, %
RF	0.0001	0.0002	0.9860	0.9575	0.90	1.04
GB	0.0001	0.0003	0.9874	0.9202	0.82	1.42
DT	0.0001	0.0006	0.9876	0.8500	0.80	1.91

### VST model prediction

Three contemporary ML techniques RF, GB, and DT were used in development of the VST ratio prediction models. After that, statistical-based assessment and comparison made advantage of the models. Table 3 lists in each scenario MAPE, R^2^, and MSE. With their identical Train MSE values and high Train R² values of about 0.97, the performance metrics for the VST ratio models reveal consistent outcomes across all three models: Random Forest, Gradient Boosting, and Decision Tree during training. This implies that every model may reasonably capture the fundamental trends in the training data. The test R² values of the models on the test data expose some variations in their generalizing capacity. With Test R² values of 0.9405 and 0.9637 respectively, the Random Forest and Gradient Boosting models show equivalent generalization. This suggests great forecasting ability despite on unavailability of data. Conversely, the Decision Tree model exhibits a somewhat lower Test R² of 0.9217, which reflects a decreased capacity to sustain accuracy on the test set, thereby maybe indicating a higher vulnerability to overfitting in compared to the ensemble-based models.

**Fig. 8:**
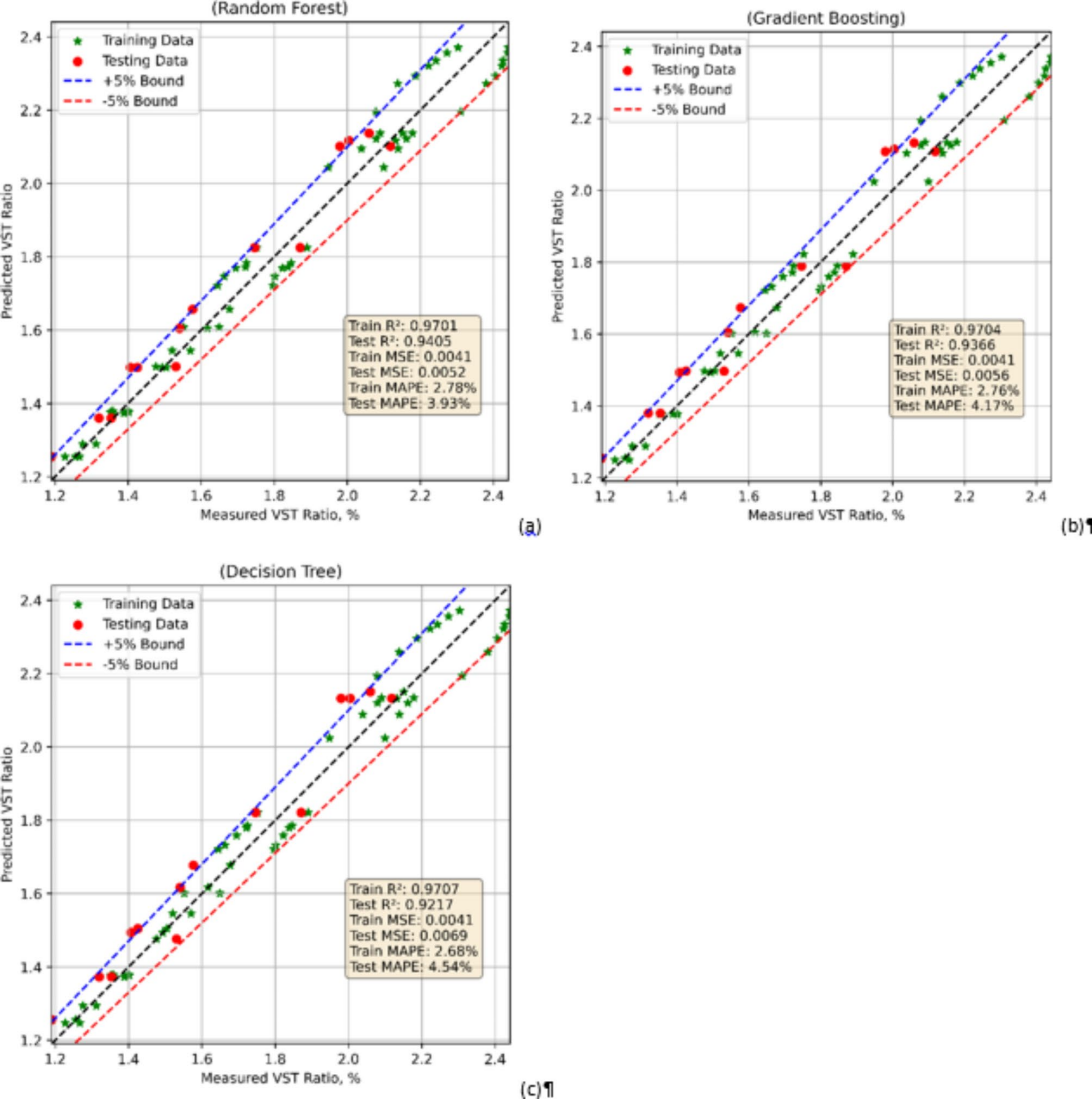
Model comparison in case of VST ratio model using (**a**) RF (**b**) GB (**c**) DT.

These trends are supported by the test data’s Mean Absolute Percentage Error (MAPE). While Gradient Boosting and Decision Tree show better error percentages, Random Forest has the lowest Test MAPE, indicating more exact predictions. This trend implies that, presumably because they have lower overfitting relative to the single-tree Decision Tree model, ensemble models especially Random Forest, handlers the volatility in the data better. Finally, all models perform well during training; with Gradient Boosting closely behind, the Random Forest model offers the best trade-off between training performance and generalization to test data. Though still useful, the Decision Tree model shows more overfitting and poor accuracy in test data predictions (Fig. 8).

**Table 3:** Model evaluations.

Model	Train MSE	Test MSE	Train R2	Test R2	Train MAPE, %	Test MAPE, %
RF	0.0041	0.0052	0.9701	0.9405	2.78	3.93
GB	0.0040	0.0056	0.9704	0.9366	2.76	4.17
DT	0.0041	0.0069	0.9707	0.9217	2.68	4.54

### eXplainable machine learning using SHapley additive exPlanations analysis

The ML approached employed in last section provided excellent results. However, these are black box methods and stakeholders may not how these models predicted or how much was the contribution of each feature involved. By pointing out the most important factors influencing TC and VST ratio predictions, explainable machine learning can offer understanding of the decision-making process of the model. Understanding feature importance and interpreting model outputs helps researchers identify causes of overfitting or errors, therefore strengthening model dependability and guiding optimization for maximum performance.

**Fig. 9:**
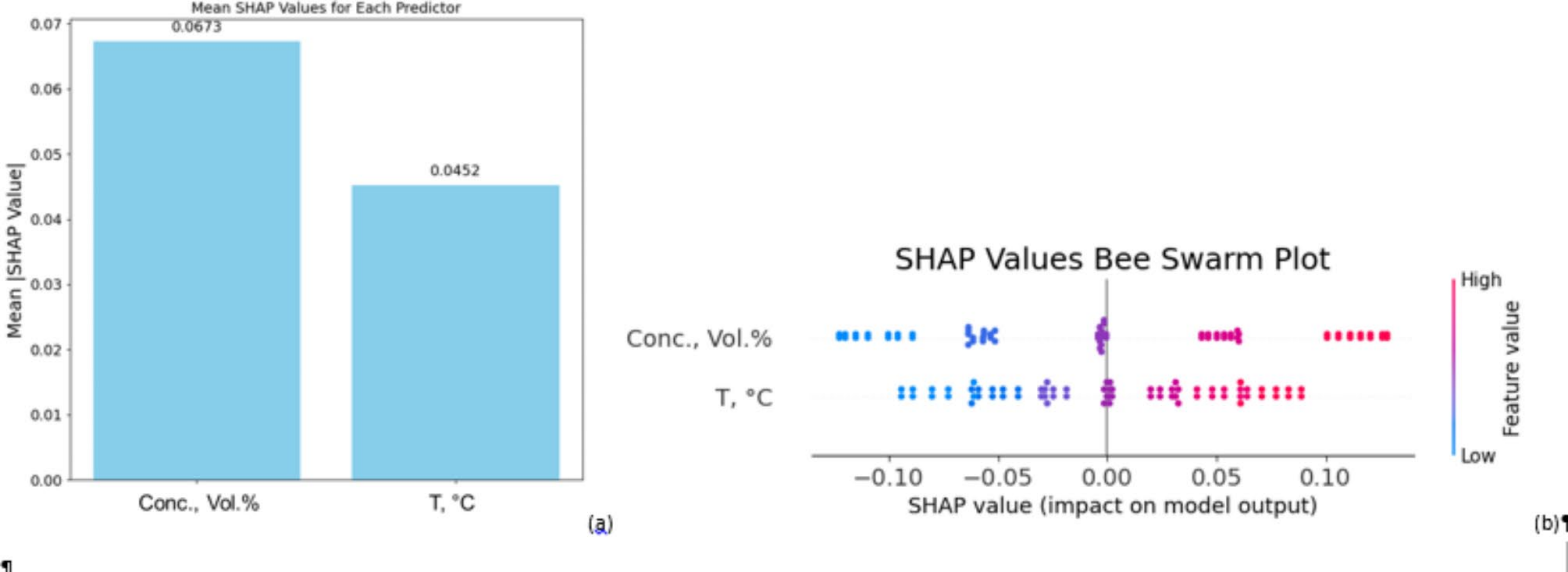
SHAP values for TC ratio model (**a**) mean values plot (**b**) bee swarm plot.

Employing SHAP values, the Fig. 9a offers a thorough examination of the relative significance of two predictors—nanoparticle concentration (Conc., Vol.%) and temperature (T, °C) on the output of the model. The first plot a bar chart of mean SHAP values showcases how mean SHAP value of nanoparticle concentration exceeds that of temperature (0.0452). This implies that, relative to temperature, concentration contributes more generally to the predictions of the model. The output shows a more significant influence from the SHAP values for concentration, so changes in concentration greatly affect the response of the model.

The bee swarm plot (Fig. 9b) expands on the distribution and range of SHAP values for every predictor. With a higher concentration of SHAP values in the positive range, nanoparticle concentration exhibits a wider distribution whereby both high and low values can either positively or negatively affect the model result. This suggests that generally the expected value rises with increasing concentration. On the other hand, temperature shows a smaller range of SHAP values; most of the values are near zero, thereby suggesting it has a more restricted influence on the prediction of the model. Though less significantly than concentration, high-temperature values nevertheless help the output in some beneficial manner. This implies that, with temperature having a minor but still significant impact, concentration dominates in determining the predictions of the model.

**Fig. 10:**
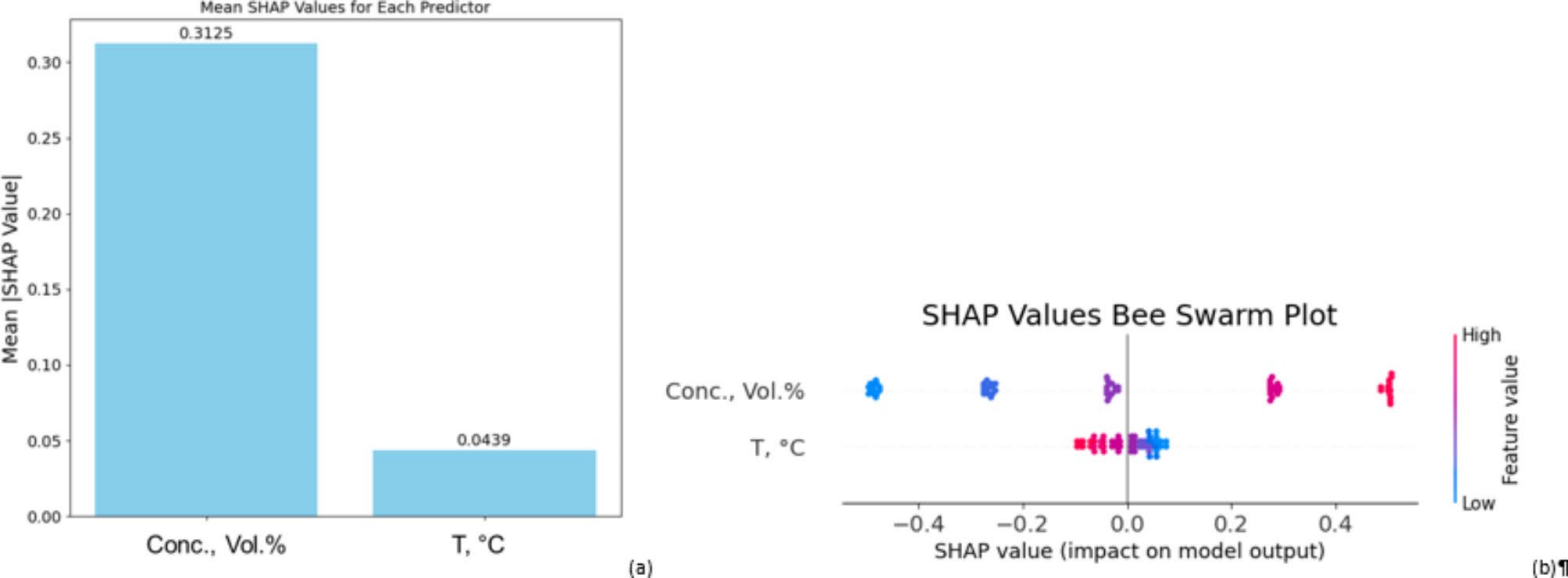
SHAP values for VST ratio model (**a**) mean values plot (**b**) bee swarm plot.

In the case of viscosity ration model, the effects of nanoparticle concentration (Conc., Vol.%) and temperature is depicted in the Fig. 10a. The bar chart shows that concentration, with a substantially higher mean SHAP value (0.3125) than temperature (0.0439), clearly influences the projections of the model. The size of the SHAP value of concentration suggests that variations in this variable cause more significant variations in the viscosity ratio prediction. With a general trend demonstrating that larger concentrations (shown by the red dots) greatly improve the model output, nanoparticle concentration shows a wide range of SHAP values both positive and negative in the bee swarm plot (Fig. 10b). On the other hand, the small range of the SHAP values for temperature indicates their reduced influence on the model since they cluster at zero. High temperatures still somewhat influence the results of the model, but less clearly. Consequently, in this model the concentration of nanoparticles still mostly determines the viscosity ratio.

## Conclusion

This study explored how temperature and concentration affect the thermal properties of various nanofluids, including GO, GO-TiO2, GO-SiO2, TiO2, and SiO2, within a concentration range of 0 to 1 vol% and temperatures from 30 to 60 °C. Using extensive experimental data, accurate prediction models were developed with RF, GB, and DT techniques. To enhance the interpretability of these complex models the SHAP (Shapley Additive Explanations) was utilized. The findings of this study offer valuable insights into the behavior of nanofluids, facilitating the creation of more effective predictive models and a deeper understanding of their thermal performance. The key conclusions are:

1. *GO* nanofluids showed the highest thermal conductivity enhancement among all tested fluids, achieving a maximum thermal conductivity ratio of 1.52 at 1 vol% and 60 °C. This enhancement is due to *GO*’s high thermal conductivity, surface area, and layered structure, which improve heat transfer efficiency.
2. Hybrid nanofluids, especially *GO-TiO*_*2*_, exhibited better thermal conductivity enhancement compared to mono nanofluids (except *GO*). At 1 vol% and 60 °C, *GO-TiO*_*2*_ achieved a TC ratio of 1.43, outperforming *GO-SiO*_*2*_ (1.39), *TiO*_*2*_ (1.23), and *SiO*_*2*_ (1.17). This improvement is attributed to synergistic effects and better nanoparticle dispersion.
3. The viscosity of hybrid nanofluids increases with nanoparticle concentration. Viscosity ratio for *GO-TiO*_2_ nanofluid at 1 vol% reached 2.45 at 30 °C, compared to 1.40 at 0.1 vol%. This increase is due to increased particle interactions and larger agglomerates that hinder fluid flow.
4. For *GO-SiO*_*2*_ nanofluid at 1 vol%, the viscosity ratio dropped from 2.30 at 30 °C to 2.07 at 60 °C. Higher temperatures enhance molecular motion, allowing the fluid to flow more freely and reducing internal resistance.
5. The Random Forest (RF) model outperformed others (Gradient Boosting and Decision Tree) in both the cases of thermal conductivity and viscosity with greater adaptability to handle fresh data during model testing.
6. Further analysis using shapely additive explanations based on cooperative game theory revealed that relative to temperature, nanofluid concentration contributes more to the predictions of the thermal conductivity ratio model. However, the effect of nanofluid concentration was more dominant in the case of viscosity ratio model.

## Data Availability

The data is available within the manuscript.
